# An Autopsy Case of Myotonic Dystrophy Type 1 With Pancreatic Intraductal Papillary Mucinous Neoplasm

**DOI:** 10.7759/cureus.70225

**Published:** 2024-09-26

**Authors:** Keisuke Nonaka, Akira Arakawa, Manato Hara, Akiko Komatsu, Takuya Nagasaka, Toshio Kumasaka, Seiya Kamino, Hirofumi Rokutan, Yuuki Shichi, Shigeo Murayama, Kazutomi Kanemaru, Chihiro Jubishi, Shutaro Futami, Toshiyuki Ishiwata, Yuko Saito, Tomio Arai, Kazumasa Harada, Joji Ishikawa

**Affiliations:** 1 Research Team for Geriatric Pathology, Tokyo Metropolitan Institute for Geriatrics and Gerontology, Tokyo, JPN; 2 Department of Neuropathology (The Brain Bank for Aging Research), Tokyo Metropolitan Institute for Geriatrics and Gerontology, Tokyo, JPN; 3 Department of Neuropathology (the Brain Bank for Aging Research), Tokyo Metropolitan Institute for Geriatrics and Gerontology, Tokyo, JPN; 4 Department of Pathology, Tokyo Metropolitan Institute for Geriatrics and Gerontology, Tokyo, JPN; 5 Department of Pathology, Japanese Red Cross Medical Center, Tokyo, JPN; 6 Department of Neurology, Tokyo Metropolitan Institute for Geriatrics and Gerontology, Tokyo, JPN; 7 Department of Cardiology, Tokyo Metropolitan Institute for Geriatrics and Gerontology, Tokyo, JPN

**Keywords:** autopsy, ctg repeat, dmpk gene, intraductal papillary mucinous neoplasm, myotonic dystrophy type 1, pancreas

## Abstract

Here, we present an autopsy case of long-standing myotonic dystrophy type 1 (DM1) in a patient who developed a pancreatic intraductal papillary mucinous neoplasm (IPMN). DM1 is a progressive genetic disorder that affects multiple organs, including the respiratory muscles. Several nationwide registry-based cohort studies have suggested that patients with DM1 have an increased risk of developing pancreatic cancers such as pancreatic ductal adenocarcinoma (PDAC). Pancreatic IPMNs are thought to progress from benign neoplasms to invasive cancers, and surgical specimens are usually required for the pathological diagnosis of pancreatic IPMNs. Although certain risk factors for developing pancreatic IPMNs reportedly overlap with those for PDAC, few cases of DM1 with pancreatic IPMNs have been reported. This is partly because pancreatectomy is associated with relatively high morbidity and mortality rates and few patients with DM1 who are suspected of having pancreatic IPMNs are candidates for surgical resection. Therefore, cases of DM1 with histopathologically diagnosed pancreatic IPMNs are rare, and the accumulation of such cases is important for understanding the association between DM1 and pancreatic IPMNs.

## Introduction

Myotonic dystrophy type 1 (DM1) is one of the most common muscular dystrophies in adults and is caused by a trinucleotide cytosine-thymine-guanine (CTG) repeat expansion in the dystrophia myotonica protein kinase (DMPK) gene [1]. DM1 is characterized by skeletal muscle weakness, myotonia, and impairment of multiple organs. Several studies have reported that patients with DM1 have an increased risk of both benign and malignant tumors in several organs compared with the general population [2,3]. Pancreatic intraductal papillary mucinous neoplasms (IPMNs) are mucin-producing pancreatic cystic tumors that arise in the main pancreatic duct and/or its branches [4]. Pancreatic IPMNs account for approximately 1% of all pancreatic neoplasms and are thought to progress from benign neoplasms to invasive cancers through DNA damage or mutation [5]. To the best of our knowledge, the association between DM1 and pancreatic IPMNs has not yet been reported. Herein, we present an autopsy case of DM1 with a pancreatic IPMN.

## Case presentation

**Figure 1:**
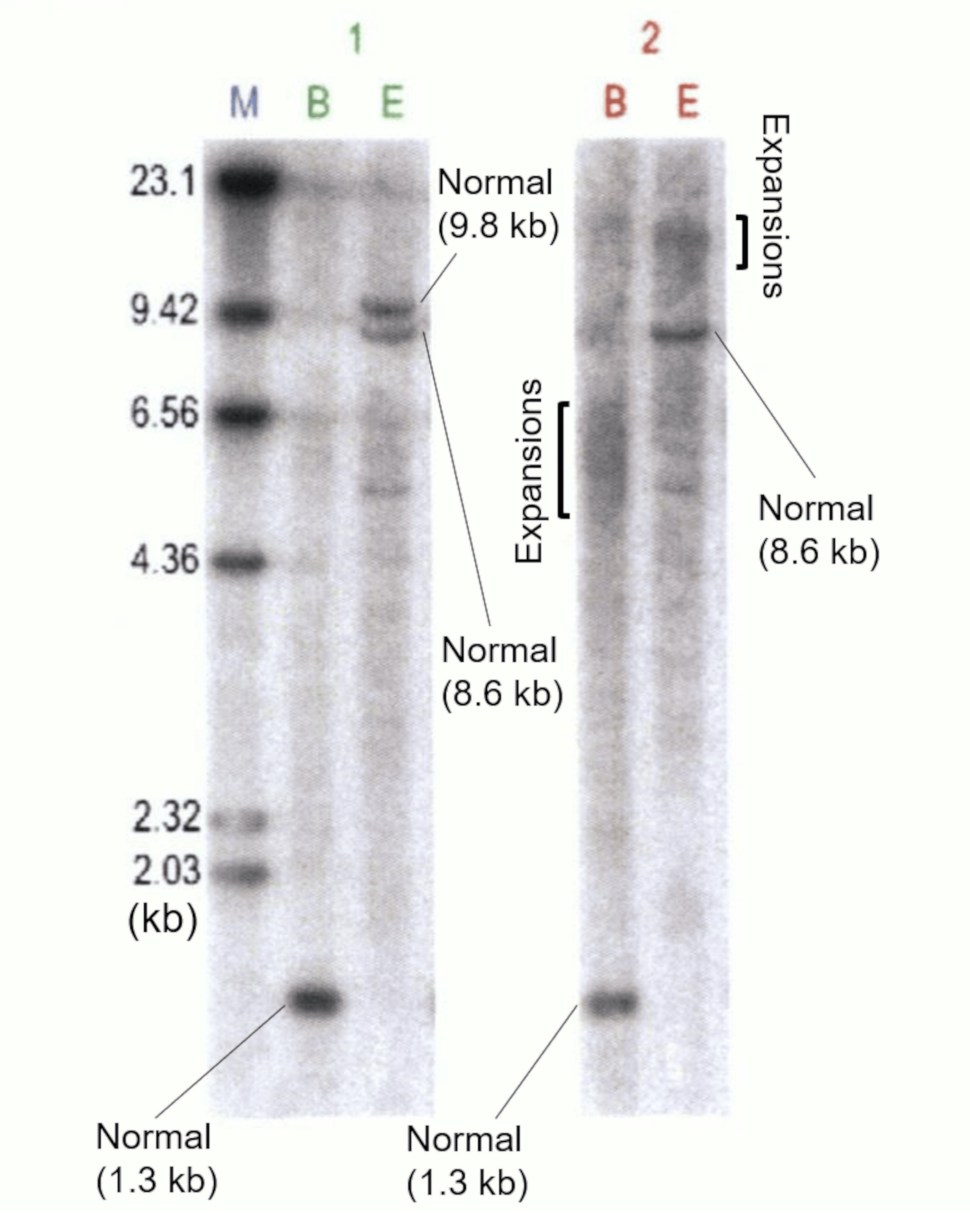
Southern blot analysis of genomic DNA probed for the DMPK gene. Genomic DNA samples were fragmented using BamH I (B) and EcoR I (E) and subjected to electrophoresis and capillary blotting to a membrane. Subsequently, the membrane was hybridized with a specific probe for the DMPK gene. Visualized fragments were from a healthy control (1) and the patient with one normal and one expanded allele (2). Estimation of the CTG repeat expansion can be done using the molecular-weight size markers (M). Normal sized alleles and expansions are indicated.

A 55-year-old woman with long-standing DM1, anemia (hemoglobin level, 9.3 g/dL), and anorexia was admitted to our hospital. During early childhood (younger than elementary school age), the patient developed skeletal muscle weakness and myotonia. Genetic testing confirmed the diagnosis of DM1, with the estimated number of CTG repeats in the DMPK gene expanding abnormally to more than 1400 repeats (Figure 1).

**Figure 2:**
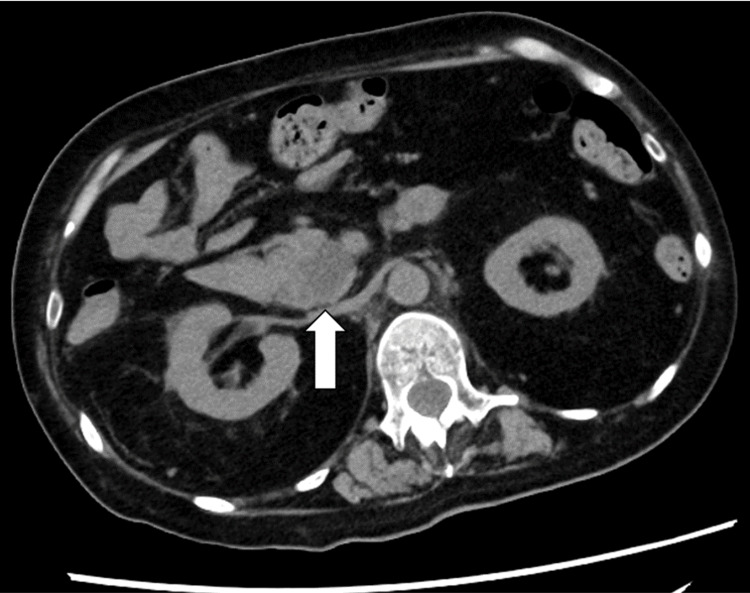
Findings of non-contrast computed tomography. A low-density area (arrow) was observed in the pancreatic head. Dilatation of the main pancreatic duct was not observed.

Although the patient’s brother was diagnosed with DM1, her parents reported no symptoms of DM1. When the patient was 41 years old, she underwent a total hysterectomy with bilateral salpingo-oophorectomy at another hospital for an ovarian tumor (the pathological diagnosis was unknown). When the patient was 46 years old, she was clinically diagnosed with pulmonary hypertension and right-sided heart failure. After the diagnosis, the patient received home oxygen therapy. When the patient was 54 years old, abdominal computed tomography (CT) revealed a 24-mm low-density cystic lesion in the pancreatic head (Figure 2) that had not been detected on a CT scan conducted seven years prior.

**Figure 3:**
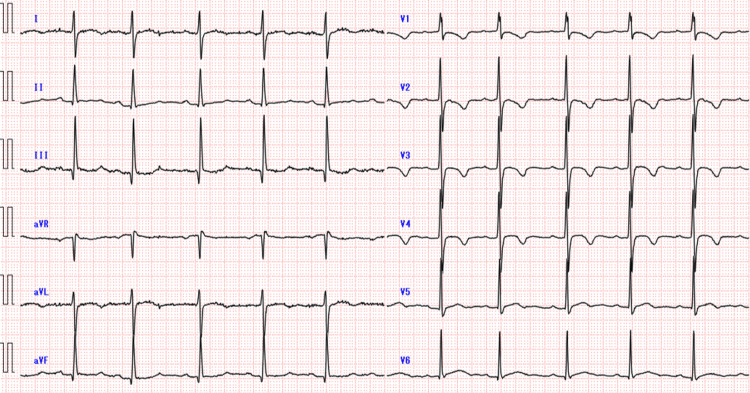
Findings of electrocardiography. An R/S ratio of greater than 1 in lead V1 and ST segment depression and asymmetric T wave inversions in leads V1 to V3 were observed.

Considering her general condition, the lesion was monitored without a diagnostic investigation. The patient was afebrile on admission. Chest auscultation revealed no abnormal findings and heart sounds indicated a regular rhythm with no murmurs. Her oxygen saturation level was 74% (3 L/min via face mask). Her blood pressure, heart rate, and respiratory rate were 89/54 mmHg, 83 beats/min, and 22/min, respectively. Electrocardiography revealed right ventricular hypertrophy, indicating a right ventricular pressure overload (Figure 3).

**Figure 4:**
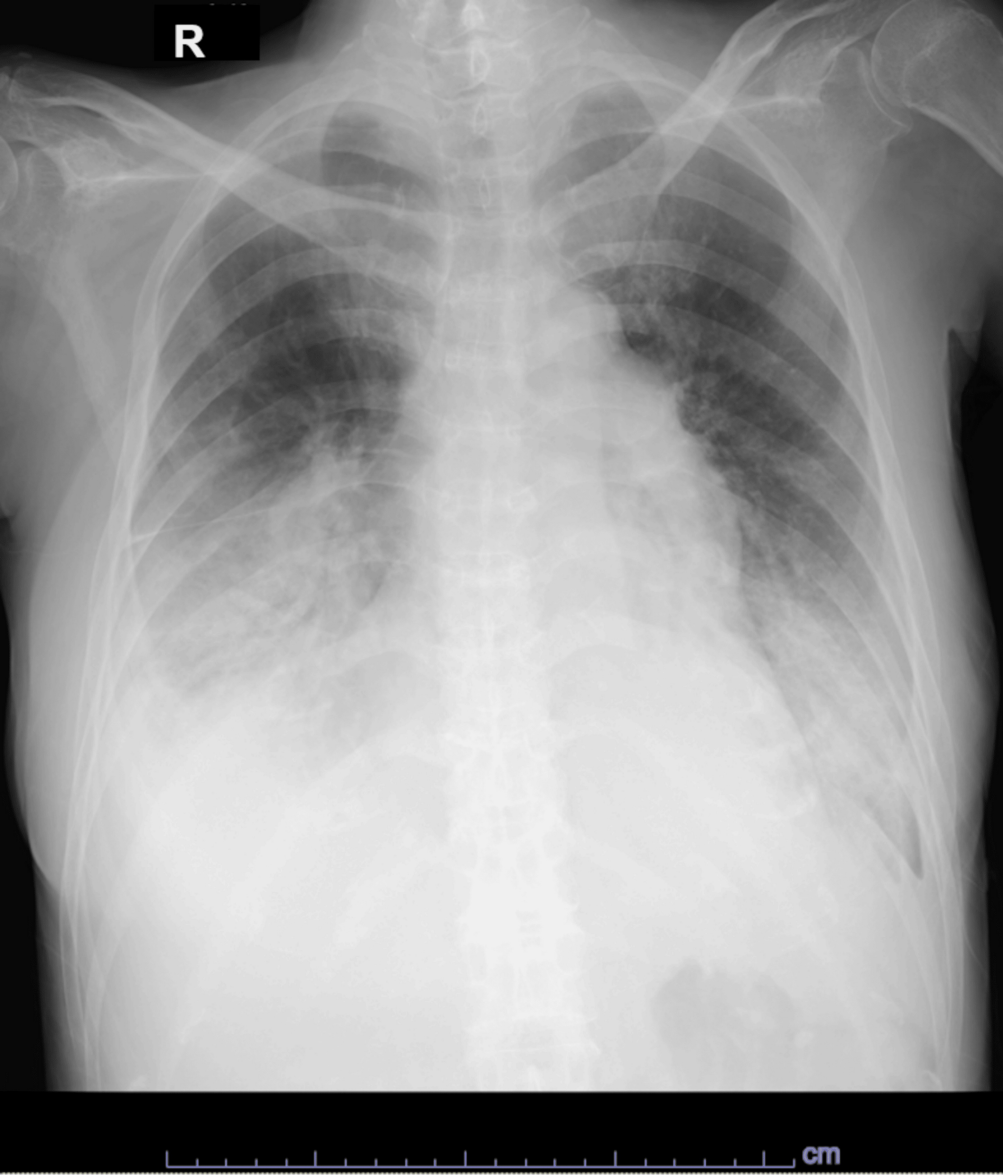
Findings of chest radiography. Bilateral pleural effusions were observed.

Chest radiography revealed bilateral pleural effusions (Figure 4), and the patient had elevated N-terminal pro-brain natriuretic peptide (3591.7 pg/mL), creatinine (1.60 mg/dL), and blood urea nitrogen (44 mg/dL) levels (Table 1).

**Table 1:** Laboratory test results of the patient upon admission. Abbreviations: WBC, white blood cell count; RBC, red blood cell count; HGB, hemoglobin; HCT, hematocrit; PLT, platelet count; BUN, blood urea nitrogen; CRE, creatinine; NT-proBNP, N-terminal pro-brain natriuretic peptide; L, lower-than-normal level; H, higher-than-normal level.

Parameters	Results	Lower limit	Upper limit	Units
WBC	6.68	3.30	8.60	10^3^/μL
RBC	3.45 (L)	3.86	4.92	10^6^/μL
HGB	9.3 (L)	11.6	14.8	g/dL
HCT	30.1 (L)	35.1	44.4	%
PLT	289.00	158.00	348.00	10^3^/μL
BUN	44 (H)	8	20	mg/dL
CRE	1.60 (H)	0.46	0.79	mg/dL
NT-proBNP	3591.7 (H)	0.0	125.0	pg/mL

These findings led to the conclusion that the patient experienced an exacerbation of right-sided heart failure. Although mild gastrointestinal bleeding was occasionally reported during hospitalization, an endoscopic examination was not performed because of her poor health condition. After discussing the treatment options with the patient and her family, she received symptomatic treatment and died on day 39.

**Figure 5:**
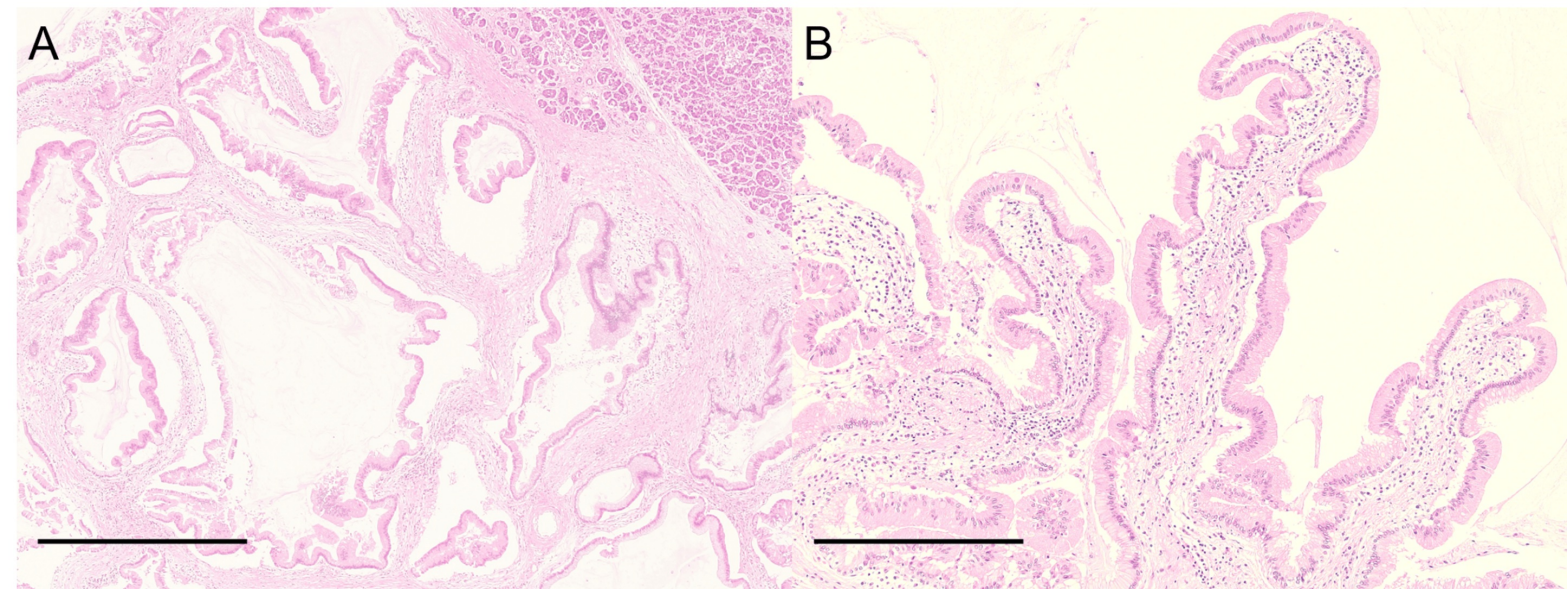
Histological images of the pancreatic head. (A) Multiple cystic lesions were observed. The normal pancreatic tissue was seen in the upper-right portion. (B) Gastric-type mucinous columnar epithelium with low-grade dysplasia exhibited a papillary growth pattern in the lining of the cystic lesion. (A, B) Hematoxylin and eosin staining. Scale bar, 1000 μm (A) and 400 μm (B).

During autopsy, three colonic polyps were detected in the ascending colon, splenic flexure, and sigmoid colon, respectively. Microscopically, these polyps were a 40-mm high-grade villous adenoma of the ascending colon and two 5-mm low-grade tubular adenomas and thought to be the cause of the gastrointestinal bleeding mentioned above. A 25-mm multilocular cystic lesion was detected in the pancreatic head. The cysts were filled with mucinous fluid. No main pancreatic duct dilation or acute or chronic pancreatitis was observed. This cystic lesion was histopathologically diagnosed as a low-grade IPMN of the gastric type arising in the branch pancreatic duct (Figure 5).

The uterus and ovaries showed the same postoperative status as mentioned above, and no local recurrence was found. No neoplastic lesions were detected in any other organs. Increased variation in muscle fiber size, an increased number of internal nuclei, and scattered pyknotic nuclear clumps were microscopically observed in the cross-section of the biceps. In the longitudinal section, the internal nuclei were often arranged in chains. These findings were consistent with skeletal muscle findings in a patient with DM1. The lungs (left lung, 465 g; right lung, 293 g) showed extensive irregular hemorrhagic foci with bilateral bloody pleural effusion (left: 150 mL; right: 550 mL). No pulmonary infarction or embolism was observed. Microscopically, small pulmonary arteries (diameter: 100-200 mm) of the lungs displayed intimal and medial thickening. Stenosis and obstruction of the small pulmonary arteries were sporadically observed. A plexiform lesion, i.e., the formation of capillary-like sinusoid channels within the native arterial lumen of the small pulmonary arteries, and a dilation lesion, i.e., dilated vein-like congestive vessels developing around the plexiform lesions, were occasionally observed. Alveolar hemorrhages were frequently observed. No inflammatory foci or hyaline membranes suggestive of pneumonia or diffuse alveolar damage, respectively, were observed. Several findings in the lungs, including stenosis and obstruction of the small pulmonary arteries, plexiform lesions, dilation lesions, and alveolar hemorrhages, indicated a severe form of pulmonary arterial hypertension (PAH). The heart (386 g) exhibited right atrial dilatation and right ventricular hypertrophy. The left atrium or ventricle did not show any abnormal findings. Pulmonary valve annular enlargement was observed. Tricuspid, mitral, and aortic valvular abnormalities, left and right atrial appendage thrombi, and significant coronary stenosis were not observed. Microscopically, both the ventricles frequently showed perivascular fibrosis. None of the findings suggested myocarditis, myocardial infarction, or necrosis. Cardiac findings, such as right atrial enlargement, right ventricular hypertrophy, and pulmonary valve annular enlargement, were consistent with right ventricular pressure and volume overload due to PAH.

## Discussion

Previous large nationwide register-based cohort studies have demonstrated that DM1 patients are at an elevated risk of developing certain benign tumors, including colorectal polyps, and specific cancers, including ovarian cancers [6-8]. In our patient, colonic adenomas detected at autopsy and surgical history of an ovarian tumor are consistent with the results of these previous cohort studies.

In DM1 patients, elevated risks of developing pancreatic cancers have been suggested in nationwide register-based cohort studies [7,8], but the risks of developing pancreatic IPMNs in DM1 patients have not been described in previous cohort studies to date. Although the pathogenesis of pancreatic IPMNs is unknown, certain risk factors for the development of pancreatic IPMNs overlap with those for pancreatic ductal adenocarcinoma (PDAC), the most common form of pancreatic cancer [9]. Furthermore, pancreatic IPMNs are frequently diagnosed during screening of individuals at high risk for PDAC [10-12], suggesting that DM1 patients may be at an elevated risk of developing pancreatic IPMNs and PDAC.

Pancreatic IPMNs are histopathologically categorized into the following three types: low-grade IPMNs, high-grade IPMNs, and IPMNs with an associated invasive carcinoma [13]. Low-grade IPMNs are benign tumors, and high-grade IPMNs are considered malignant. Surgical specimens are typically required to pathologically diagnose these three IPMN categories. In clinical practice, several guidelines exist for the diagnostic and therapeutic management of pancreatic IPMNs [4,5]. Diagnostic investigations are performed to select patients indicated for surgical resection. Respiratory dysfunction is the most common cause of death in DM1 patients [1]. In addition, pancreatectomy is a complex procedure that is associated with high morbidity and mortality rates [4]. This suggests that DM1 patients with a suspicion of having pancreatic IPMNs are rarely indicated for surgery, and surgical pathology data on pancreatic IPMNs in DM1 patients may be rarely available in nationwide register-based cohort studies.

In this patient, the number of CTG repeats was relatively large (>1000 repeats). In general, a larger number of CTG repeats correlates with an earlier age of onset of symptoms and more severe disease [14]. In addition, the decrease in muscle strength in DM1 patients is highly correlated with disease duration and the number of CTG repeats [15]. On the other hand, no correlation between the length of CTG repeats and tumor development has been observed in patients with DM1 [2,16], suggesting that the number of CTG repeats would not be associated with the risks of developing pancreatic IPMNs.

The autopsy disclosed PAH, a rare, progressive lung disease [17]. To our knowledge, an association between DM1 and PAH has not been reported to date. PAH affects the small pulmonary arteries and increases the right ventricular afterload, resulting in right-sided heart failure. PAH is caused by various etiologies or triggers, such as idiopathic PAH and PAH associated with connective tissue disease [17]. According to the subgroup classification of PAH by the World Symposium, our patient with DM1 had no triggers or diseases associated with PAH, suggesting that our case developed idiopathic PAH.

## Conclusions

Our patient with DM1 had a low-grade gastric-type IPMN in the pancreatic head. Although the risks of developing pancreatic IPMNs in DM1 patients have not been described in large nationwide cohort studies, these studies have suggested that patients with DM1 are at an elevated risk of developing pancreatic cancers, such as PDAC. As the risk factors for the development of pancreatic IPMNs overlap with those of PDAC, patients with DM1 may be at an elevated risk of developing pancreatic IPMNs. Additional research and accumulation of autopsy cases are necessary to clarify the association between DM1 and pancreatic IPMNs.
